# Strategies to maintain health service provision during the COVID-19 pandemic in refugee settings in Jordan and Uganda

**DOI:** 10.1371/journal.pgph.0004484

**Published:** 2025-05-08

**Authors:** Gabrielle Prager, Heba Hayek, Muhammad Fawad, Ronald Nyakoojo, Julius Kasozi, Adam Musa Khalifa, Paul Spiegel, Chiara Altare

**Affiliations:** 1 Johns Hopkins Bloomberg School of Public Health, Baltimore, Maryland, United States of America; 2 Johns Hopkins Center for Humanitarian Health, Baltimore, Maryland, United States of America; 3 United Nations High Commissioner for Refugees, Amman, Jordan; 4 United Nations High Commissioner for Refugees, Kampala, Uganda; Georgetown University, UNITED STATES OF AMERICA

## Abstract

Health system adaptations were rapidly introduced at the start of the COVID-19 pandemic to protect the public and maintain access to health services. Given the specific vulnerabilities of forced displacement settings, understanding which adaptations were used, how they were implemented, their success, and challenges is important for preparedness and response efforts. In this paper, we characterize adaptations in health service delivery implemented by Ministries of Health, the UN Refugee Agency, and partners to maintain health services provision for refugees in Jordan and Uganda. We conducted 21 key informant interviews with managerial and operational staff across 12 organizations who delivered healthcare services for refugees in Uganda and Jordan during the COVID-19 pandemic and applied a framework analysis to the adaptations characterized. The results are presented by WHO health system building blocks. Most adaptations focused on health service delivery specifically procedures for screening and isolation in the community, COVID-19 community support, and facility-level infection prevention measures. Health service delivery adaptations focused not only on ensuring capacity for COVID-19 patients but on adapting mechanisms to support access for those needing regular care. Many adaptations worked in tandem with others as packages to achieve this. Workforce adaptations included task shifting and staffing surges. Modifications related to medical products, vaccines, and technologies focused on procurement, medication management, supporting vaccine strategies, and building testing capacity. Adaptations in leadership and governance, financial and health information systems were identified but mainly described as essential enablers for other adaptations. Key enablers to successful adaptation in this context included the integration of refugees in National Health systems, strong relationships between partners and a supportive environment for adaptation, existing preparedness plans and access to financing. This study highlights the scale, scope and diversity of innovative adaptations implemented to maintain health services for refugees in Jordan and Uganda during the COVID-19 pandemic.

## Author summary

COVID-19 saw the rapid introduction of health system adaptations to maintain access to health systems. These adaptations helped protect patients from the virus as well as support access to existing care, minimizing interruptions. This research summarizes the adaptations introduced in refugee settings in Jordan and Uganda to support documentation and learning for future outbreaks. We highlight the breadth of adaptations described to us by staff responsible for their introduction and application. Adaptations were numerous and implemented across all aspects of the health system. They included more universal infection control adaptations and those specific to camp and settlement settings to protect new arrivals, as well as mechanisms to ensure patients could continue to access routine care through pregnancy and for long-term illnesses, for example. The longstanding relationships between partners and the government, as well as the integration of refugees in national health systems, were fundamental to fostering an adaptive environment that supported the COVID-19 health response.

## Introduction

The first COVID-19 case was identified in Wuhan, China, in December 2019 [1]. By the end of 2023, over 770 million cases and 7 million deaths were reported worldwide, with varying spread and speed across countries and contexts [1,2]. Humanitarian and forced displacement settings have particular vulnerabilities, including crowded living conditions, limited space, lack of adequate sanitation and water, reduced access to health care, and a considerable reliance on donor funds [3,4], that were feared to increase the risk of spread and impact of COVID-19 [3,4]. Yet disruptions were delayed and more limited in time and scale than expected. Infections have spread extensively, and excess mortality has been reported [5,6]. Analysis from the first year of the pandemic in Uganda has shown lower COVID-19 incident rates in camps and settlements than amongst the general population, with similar rates in Jordan, and initial but temporary disruption to existing routine health services [7,8]. However, the impact has not been comprehensively captured with subsequent waves of increasing cases. Routine health services have been negatively affected by previous epidemics in humanitarian settings, diverting supplies and exacerbating existing health system challenges such as staff shortages. Associated lockdowns and restrictions further worsen displaced populations’ access to care [9]. COVID-19 brings specific risks to those with chronic conditions, particularly in humanitarian settings where follow-up care is limited, and ensuring the continuation of services for this vulnerable group poses further challenges [9].

From the outset, Ministries of Health (MoHs), United Nations (UN) agencies, and health actors quickly recognized the need to adapt health service delivery to protect users and maintain function. Adaptations were swiftly developed and implemented, and guidelines were compiled by national authorities, the UN, and individual organizations to support the response. The situation evolved rapidly, and context-specific adaptations emerged. The documentation of context-specific adaptations implemented by health actors occurred in various fragmented ways, including the COVID-19 Humanitarian platform [10,11], a repository of guidance documents and qualitative interviews [11,12], and thorough individual studies. For example, Klabbers et al. investigated the willingness to adopt prevention measures, barriers, and facilitators of COVID-19 risk mitigation strategies as part of “Dial-COVID”, a toll-free line for symptom surveillance and information dissemination in refugee settlements in Uganda [13]. Evens et al. documented adaptations and COVID-19 prevention strategies to family planning and reproductive health services in Nigerian humanitarian settings in the first year of the pandemic [14]. Ratnayake et al. analyzed the changing role of community health workers (CHWs) and adaptations for maintaining hypertension and diabetes care among Syrian Refugees in Jordan [15], and Miller et al. reported program adaptations to support people with non-communicable diseases (NCDs) across countries where the International Rescue Committee implements health programs [16].

Besides the COVID-19 Humanitarian platform and the few studies mentioned, the range and breadth of adaptations in refugee settings across the health system have not been documented in detail. Understanding which adaptations were used, how they were implemented, their success, and challenges can inform preparedness and response strategies to future outbreaks in forced displacement settings. To that end, we aimed to characterize adaptations in health service delivery modalities and strategies implemented by the MoH, the United Nations High Commissioner for Refugees (UNHCR), and partners to maintain health services provision for refugees; identify which adaptations were most effective and accepted, as well as which modifications should be continued after and beyond the context of COVID-19.

## Methods

### Ethics statement

This work was deemed Non-Human Subject Research by the Johns Hopkins Bloomberg School of Public Health’s Institutional Review Board (Notice n 23096). All respondents participated in their professional capacity and after providing oral consent.

### Study design

We present here a qualitative study that employed key-informant interviews to identify and describe COVID-19 adaptions employed to maintain health system services for refugees. It forms part of a broader project aimed at understanding the strategies to maintain health service delivery among refugee populations in and out-of-camp and settlement settings in Uganda and Jordan. The project encompassed three components, the first of which is reported here. The other components include an interrupted time series analysis of routine health data to assess changes in health service utilization and outcomes and a household survey and focus group discussions to understand community perspectives on program adaptations, access to health services, and healthcare-seeking behavior during the COVID-19 pandemic.

### Study settings

Uganda is the largest refugee-hosting country in Africa [17], with approximately 1.6 million refugees (as of the end of 2023), of which 56.6% originate from South Sudan and 31.1% from the Democratic Republic of Congo [18]. Refugees predominantly live in settlements, and approximately 8% live in the capital, Kampala [17]. The first case of COVID-19 in Uganda was reported on March 20th, 2020, and the first case in a refugee settlement was in Adjumani on May 22nd, 2020 [7]. Approximately 170,544 COVID-19 cases were reported in Uganda by March 2023 [19]. By the same period, a total of 90,206 refugees had been tested for COVID-19, of which 7,332 tested positive [20].

Jordan hosts approximately 720,000 refugees registered with UNHCR (as of the end of 2023), 90% of whom are from Syria and, unlike Uganda, mostly live outside of refugee settlements or camps [21]. Zaatari and Azraq refugee camps hosted about 80,000 and 40,000 Syrian refugees, respectively, at the end of 2023 [22]. The first case of COVID-19 in Jordan was reported on March 2nd, 2020, and the first cases in Azraq and Zaatari refugee camps [8,22] were reported on September 8^th^ and 13^th^, 2020 [8,22]. Approximately 1.7 million cases of COVID-19 had been reported in Jordan by the end of 2022 [8]. As of March 2023, 6,768 COVID-19 cases had been reported across Azraq and Zaatari refugee camps, 4,263 in Zaatari and 2,505 in Azraq [23].

### Study participants and sampling strategy

The study aimed to document and assess the broad range of adaptations employed by various organizations in each country, in and out of refugee camps/settlements, and at multiple levels across the health system. A purposive sample of individuals working in managerial and operational roles across UN agencies and non-governmental organizations (NGOs) who worked in the delivery of healthcare services in each country during the COVID-19 pandemic was collated. Invitations for interviews were extended to individuals via email between February and March 2023.

### Data collection

A semi-structured, in-depth interview guide was developed collaboratively by the study team to explore adaptations in healthcare services over time throughout the COVID-19 pandemic (S1 Text). A simplified interview guide was shared with participants in preparation for the interviews to facilitate recalling changes over time (S2 Text). Two authors (CA and GP) conducted the interviews over Zoom between February and April 2023 (approximately 1-1.5 hours). All respondents provided informed consent. Notes of the interviews were taken contemporaneously into the interview guide, and interviews were recorded and revisited for clarification.

### Data analysis

**Fig 1:**
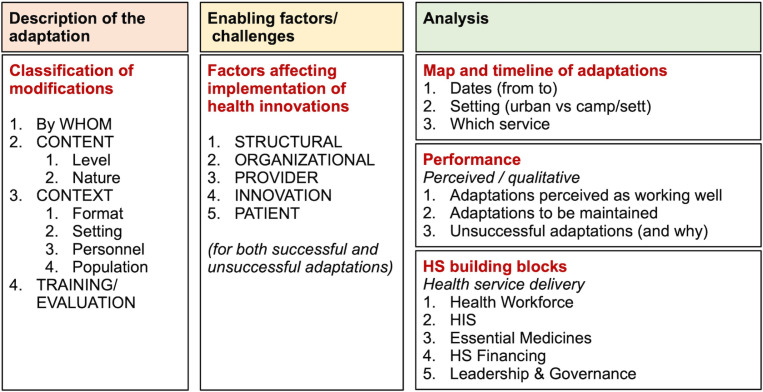
Analysis framework for COVID-19-related adaptations introduced in refugee settings in Jordan and Uganda. Adapted from Stirman et al.’s System of classifying adaptations and modifications, Chaudoir et al.’s multi-level framework predicting implementation outcomes and the World Health Organization’s Heath system building blocks. [24,25,27].

Drawing on Striman’s work on the classification of modifications made to evidence-based interventions [24] and Chaudoir’s on factors affecting the implementation of health innovations [25], we based our analysis on the conceptual framework depicted in Fig 1.

We applied the framework method to analyze the interview data, enabling a systematic qualitative data analysis [26]. The unit of analysis was one individual adaptation captured per row of the framework. For each adaptation, we extracted the following variables from the interviews: description of the adaptation (who made the adaptation, what was adapted, and the level and nature of the adaptation), the location where adaptations were carried out (in or out-of-settlements/ camps), challenges and enablers in implementation, community and organizational opinion about their perceived performance and the adaptations developments over time, this was performed and recorded in Microsoft Excel (v16). Adaptations were then consolidated and organized by the WHO’s health system building blocks [27].

## Results

We interviewed 21 individuals across 12 organizations. Participants held various field managerial and technical roles. Table 1 demonstrates the breakdown of participants by organization type, role, and location.

**Table 1:** Participant organization by type, work setting, and role, Jordan and Uganda, 2023.

	Jordan	Uganda
**Total**	**13**	**8**
**UN agencies**	**8**	**2**
*Urban*	*2*	*–*
*Camp/ Settlement*	*3*	*–*
*Nationwide*	*3*	*2*
**NGO**	**5**	**6**
*Urban*	*1*	*1*
*Camp/ Settlement*	*3*	*3*
*Nationwide*	*1*	*2*
Position and Role *		
Managerial	4	7
Technical	7	3
Field	4	3

Note: *Position and role were not mutually exclusive

The interviews provided insight into the large number and variety of adaptations organizations used to maintain health services for refugees during the COVID-19 pandemic in these settings. Adaptations occurred throughout the health system and are presented below by the World Health Ogranization’s (WHO) health system building blocks [27]: Health service delivery; workforce adaptations; medical products, vaccines, and technologies; health information system; financing adaptations; and leadership and governance. S1 Table presents a table summary of the adaptations discussed.

### Health service delivery adaptations

Most adaptations were in health service delivery and as the majority of adaptations described designed to support and implement effective infection prevention and control (IPC) across the community and facilities while maintaining services (Table 2). These varied by context and organization but were centered around the following three themes that were divided into community and facility-level measures:

i) Procedures for screening and isolation in the community
ii)Facility-level infection prevention measures
  a. Facility organization;
  b. Case management and referrals;
iii) COVID-19 community support.

The hierarchy of controls is a framework frequently used to support comprehensive IPC implementation [28–30]. Community-level measures addressed the elimination and substitution of COVID-19. Elimination and substitution aim to remove or replace the hazard from health facilities and are identified as the most effective areas for implementation for IPC [28,30]. Facility-level infection prevention measures, include engineering and administrative controls [28,30]. Engineering controls refer to design measures taken to reduce transmission through healthcare facility design, and administrative controls are the measures taken to reduce infections among staff and patients in health facilities through policies, procedures, and training [28,30]. These have been presented together as often facility reorganization and design were accompanied by policy changes. Many of the workforce and pharmaceutical adaptations were also administrative or engineering controls adaption in nature but kept separate by health system building block as this was the main framework for our analysis.

### Procedures for screening and isolation in the community

Reducing infection transmission in camp and settlement settings was challenging, particularly with new arrivals. In Uganda, one strategy to reduce new infections was zoning, where groups of new arrivals split into cohorts (i.e., all people who arrived together formed a cohort) to complete a period of isolation together. Once testing was available, the cohort members were tested together. In the event of a positive test, only the cohort was required to continue isolation in the transit center and not the entire group arriving in the camp. This measure was perceived as helping instill trust and reducing security risks, as it reduced the length of time new arrivals needed to isolate.

In Jordan, separate isolation and quarantine areas were built within camps where isolating individuals with positive tests could access food, sanitation, and other facilities. Before this approach was introduced, one entire plot would be isolated if a person tested positive, but as cases increased, this was reduced to household-level isolation. Toilets and kitchens were also built to support household-level isolation and reduce the need for plot-level isolation. Throughout this period, households were assisted with food parcels, hygiene kits, etc. Still, the move to isolate in pre-established areas helped support individuals needing to quarantine. As Rapid Diagnostic Tests (RDTs) for COVID-19 became available and the reliance on polymerase chain reaction (PCR) tests reduced (often with long wait times for results and uncertainty while these results were awaited), the organization and facilitation of isolation became more straightforward. Hotlines were made available for those isolated in the community to provide information or assistance.

### Facility-level infection prevention and control measures

#### Facility organization.

Infection prevention and control (IPC) adaptations were the most mentioned adaptations and included mandatory handwashing, facemask wearing, and physical distancing in waiting rooms, clinic spaces, and wards. Cleaning efforts intensified. Clinic opening hours changed, and the time between appointments increased to allow for more thorough cleaning. Specific patient pathways were developed to support patient flow and ensure suspected COVID-19 patients were kept separate from those who were not. Separate waiting rooms for patients with respiratory symptoms or fever were also developed. Visitor restrictions were also introduced to reduce crowding. COVID-19-specific patient transportation or ambulances were also designed to limit cross-infection of patients (see above). In Uganda, IPC volunteer roles were created to support temperature screening and ensure mask-wearing and physical distancing for arrivals at the health center. Organizations described most adaptions of the clinical physical space through designing additional waiting spaces and building isolation units. In Uganda, some facilities reported moving clinical consultations outside for improved ventilation. In Jordan, one facility reported using a caravan to create a separate space for testing.

To reduce in-person interactions at the health facility, the number of patients seen daily in the clinic was often lessened through clinic hours reductions and changes. Specific services (antenatal and postnatal care, for example) were moved to the community where possible in both countries. In Jordan, certain services (for example, dental services, nutrition, and newborn screening) were de-prioritized and subsequently reopened with a catch-up process. Infant and young child feeding counseling was initially stopped due to the small number of people using the service, but eventually, it was restarted with greater community outreach for screening. In Uganda, antenatal care services were reduced to monthly services with outreach for high acuity cases. Care for chronic conditions, particularly for stable patients, was frequently shifted to telemedicine and CHWs’ visits. Unstable or new patients would still visit the health centers. This was accompanied by an increase in prescription length, detailed below. Telemedicine took various forms in both countries and included hotlines for advice on existing conditions or COVID-19. Rarely was this in the form of entire consultations conducted over the phone due to limited phone and data access. Patients in both contexts were reported to prefer being seen face-to-face.

In Jordan, appointment systems were introduced in some locations to reduce congestion, with walk-in appointments available only for urgent cases. The appointment system was challenging to implement as many expected to be seen as they had been historically, and flexibility was necessary at the outset. The appointment system remains in use.

Triaging needed adaptation to identify and separate suspected cases and prioritize those needing immediate care. In both countries, temperature screening on arrival was used to isolate patients before further assessment. In Uganda, fever was not specific enough due to the prevalence of malaria, and differentiating causes of fever was challenging until COVID-19 RDTs were available. Malaria RDTs were incorporated in triage to support this differentiation. One facility moved Malaria RDTs to consultation rooms to reduce patient movement to the laboratory and limit the exposure of other patients to potential cases.

#### Case management and referral redistribution.

In both countries, selected governmental health centers and hospitals treated severe cases. COVID-19-specific areas for care, treatment, and isolation were developed locally. COVID-19 ambulances were designated to move patients to local health facilities or specific facilities for severe cases in both countries. In Jordan, CHWs were tasked with following up with cases to ensure they were isolating, delivering support, and identifying patients needing higher-level care. Telemedicine was introduced in one quarantine location to facilitate follow-up with cases and limit healthcare staff exposure. COVID-19-specific treatment centers were built in camps as extensions to health facilities and remained in case of future outbreaks.

In Uganda, all COVID-19 patients were initially referred to hospitals in Kampala, but as cases grew, subregional centers were identified to care for COVID-19 cases. When centers experienced rapid increases in COVID-19 patient volume, one organization supported patient redistribution across their centers to ensure adequate oxygen availability. A hotline was available for health facility staff who could request support managing acute COVID-19 cases locally. MoH guidelines for treating mild cases were also adapted; mild cases were treated in the settlements and isolated at health facilities to limit spread in the settlements. Later in the pandemic, home-based treatment models were introduced, with Village Health Teams (VHT) (CHWs are organized in VHTs in Uganda. We will refer to CHWs/VHTs in the paper to refer to community members tasked with the provision of health services at the community level.) visiting households and some conducting RDTs.

All-cause non-acute referrals were de-prioritized in both countries to reduce patient volume at the secondary and tertiary levels. In Jordan, specific MoH hospitals were also designated to receive acute cases of non-COVID patients to maintain services. Existing referral mechanisms relied on patients submitting documentation for reimbursement in person at the health center. WhatsApp lines were set up to submit the same documentation without visiting the health facility, reducing crowding. This innovation was well received and continued.

Some ‘reverse’ referrals were instituted in Uganda, where specialized physicians would visit lower-level centers to support managing patients with complex or specific non-COVID-19 related needs. The reverse referrals reduced the burden on tertiary-level centers and were used for dentistry, ophthalmology, and gynecology.

In both countries, transport to and from health centers posed a challenge. In Uganda, several organizations provided additional ambulances and repurposed vehicles to support patient transportation. Priority was given to severe cases, but also pregnant women, children, older persons, and those in villages furthest from health facilities. In Jordan, a mobile clinic with a driver, doctor, and nurse was established in one camp to care for acute conditions. They treated patients in the community as much as possible to limit the potential exposure at the health center; patients could also be flagged by health care personnel for follow-up by the mobile clinic.

#### COVID-19 community support through engagement, messaging, and surveillance.

COVID-19 community support was implemented in various forms. COVID-19 messages on COVID-19 risk, symptoms, prevention, health service changes, and vaccination were disseminated through flyers, social media, and CHWs/VHTs. CHWs were trained in health awareness and hygiene measures, which were integrated into their home visit protocol. In Jordan, messages highlighted the positive impacts of increased IPC, including “protecting loved ones”, which were reported as well received. In Uganda, toll-free lines were developed for individuals to gain information about COVID-19 and its prevention and request medical support. Boda-Boda talk-talk drove around, and VHTs walked with megaphones disseminating COVID-19 awareness and prevention messages.

CHW and VHTs conducted surveillance activities in camps and settlements. In Uganda, helplines for surveillance communication were developed for community leaders and VHTs. VHTs identified potentially unwell individuals at home and organized for transport to the health facility by ambulance or repurposed vehicle. In some localities, VHTs could request mobile medical teams to visit and treat sick individuals in their homes. VHTs were also capacitated during later waves to treat mild and moderate cases at home. VHTs had leads in case management or infection control whom they could contact in case of any questions.

In Jordan, psychosocial support services were delivered over the phone during the lockdowns, and WhatsApp groups were used to support service users who were sick with COVID-19. Cash for health services had to identify new means of delivering cash, including setting up online e-wallet accounts beneficiaries could register for to receive electronic payments and expanding existing iris-scanning schemes to allow registered users to withdraw cash without face-to-face contact; for those particularly high-risk, money was delivered directly to these individuals by car.

In both countries, CHW/VHT services were stopped initially but recommenced quickly with IPC measures and guidance, including mask and glove wearing, more concise information delivery, and door-to-door instead of group messaging. As lockdowns eased, the role of CHWs was further adapted to include visits to ensure families received follow-up due and re-link individuals to services.

**Table 2:** Detailed examples of adaptations implemented by organizations across health service delivery.

Adaptation theme	Explanation	Country examples
**Health service delivery adaptations**		
*Procedures for screening and isolation*	Facility- and camp-level processes to screen and isolate potential cases.	**Jordan** • Defined areas in camps for isolation and quarantining, with separate services including food, sanitation • Quarantine area near health facility with monitoring from the health facility for more severe cases. • Plot-based isolation for community cases in camps. • Introduced individual kitchens and latrines for households. • Hotline for isolating individuals needing support. • House-to-house surveillance. **Uganda** • Zoning: cohorting new arrivals in smaller transit centers for joint quarantine. • Community identification and screening of new arrivals. • Isolation units built for suspected cases. • Toll-free line for VHTs and community leaders to support surveillance communication.
*Facility-level infection prevention and control measures* •*Facility Organization*	Processes introduced at facility levels to reduce to reduce risk of contact transmission and free up health care workers to manage COVID-19 cases. This includes changes to triage processes.	**Both countries** • Provided masks to patients and visitors. • Limited visitors to health facilities. • Reorganized waiting areas, new seating for physical distancing, separated waiting areas for suspected cases. • Earlier opening, later closing and increased time between appointments for cleaning. • Dedicated clinic spaces for patients meeting the case definition. • Increase handwashing facilities. • Increased spacing between beds. • Multi-month prescribing (NCD, TB, HIV, Nutrition, FP). • Health care providers conducting telephone consultations for stable patients with chronic illnesses (NCD and some gynecological care). • Outreach mobile care for ANC and PNC. • Home visits for high-risk, unstable, or newly diagnosed patients with chronic illnesses. • Temperature screening and case definition screening on arrival to health facilities. **Jordan** • Dedicated facility pharmacies for suspected cases. • Staff to manage crowds at the health facilities. • Different entry and exit points in the facility for suspected cases. • Random staff testing. • Mobile teams for acute cases, cases flagged by CHWs. • Positive cases telephone follow-up. • Hotlines available for NCD patients to receive advice. • Appointment system introduced. • Patient transportation system to facilitate movement to the clinic. • Limited certain services, including dental care, newborn screening, growth monitoring. • Reduction of patients seen in the clinic per day. • Community outreach by CHWs to NCD patients. • Telephonic psychological support services. • Separate secondary triage for suspected cases. **Uganda** • IPC volunteers recruited and trained to institute and maintain IPC measures at facilities. • Earlier clinic opening, to reduce morning crowds. • Limited clinic time to half a day. • Reduce face-to-face appointment frequency for clinically stable patients with chronic illnesses. • Reduced frequency and contact time of house-to-house VHT visits. • Malaria RDTs moved to consultation rooms/ isolation areas to identify other causes of fever and limit patient movement to the laboratory. • Identified isolated beds ready to care for critically unwell COVID-19 patients on arrival.
*Facility-level infection prevention and control measures* • *Case management and Referral pathways*	Referral mechanisms to provide care for COVID-19 patients, reduce referral burden on secondary and tertiary care level facilities, as well as maintain care for patients that would have ordinarily been referred	**Jordan** • Designated MoH facility for urgent non-covid referrals, new referral mechanisms into MoH facilities for non-COVID-19 cases. • WhatsApp service to upload requests for and receive reimbursements for MoH facility care. • Newly built specific COVID-19 treatment centers to manage cases. **Uganda** • Toll-free number for COVID-19 treatment advice and referral from local facilities to designated COVID-19 secondary care facilities. • Delayed cold-case referrals to tertiary centers. • Reverse referrals, specialists visited the lower-level health centers. • All COVID-19 patients initially referred to the capital, subsequently to a subregional center. • Redistributed patients regionally to manage Oxygen availability. • COVID-19-specific ambulances: to pick up patients in the community and transfer them to treatment centers.
*COVID-19 community support*	Methods to inform the public about COVID-19 and to support health care seeking in the community	**Both countries** • COVID-19 advice hotlines. **Jordan** • Social media messaging on COVID-19 and health facility changes. • Target CHW visits to community members vulnerable to COVID-19. • Posters and flyers. • Change in cash for health services with greater routes for accessing funds including the introduction of e-wallets, expansions of Iris scanning schemes and home delivery. • CHW delivery of supplies such as masks along with messaging. **Uganda** • Repurposing other vehicles for health to collect and bring community members to the health facility. • VHTs met the public in small groups to explain Health System changes, IPC, and COVID-19. • Recorded health messages disseminated through speakers on motorbikes. • VHTs with megaphones to disseminate information.

*Acronyms used in the table: CHW: community health worker, VHT: Village Health Team, RDTs: Rapid Diagnostic Tests, MoH: Ministry of Health, NCD: Non-communicable diseases, TB: Tuberculosis, HIV: Human Immunodeficiency Virus, FP: Family Planning, ANC: Antenatal Care, PNC: Postnatal care, IPC: Infection Prevention and Control, PPE: Personal Protective Equipment,*

### Workforce adaptations

Most workforce adaptations had three main goals: i) surge staff availability to care for increased COVID-19 patient load; ii) reduce non-COVID-19 referrals out of the clinic to avoid overburdening higher-level facilities; and iii) maintain services despite staff illness or need to quarantine (Table 3). Adaptations included new clinic hires, changes to clinic hours and shifts, reduced capacity building, and the interruption of non-acute services to redistribute staff to support acute care. The challenge of maintaining staffing and services was significant.

In Uganda, surge teams were recruited to fill gaps and increase staff-patient ratios. Staff were often junior and recruited in roles outside their core competencies. Training and mentoring schemes were developed to support them. Burnout was common as staff could not leave the health facility or take leave for extended periods. In one example, surge teams were brought in later in the pandemic to allow staff to take leave and visit family. One organization also supported staff living at the health facilities who became unwell, assisting with supplies such as groceries. Such support was not frequently mentioned in the interviews.

In Jordan, several adaptations took place anticipating the lockdowns and the challenges staff might face when traveling. Pre-existing strong relationships between UNHCR and the MoH facilitated obtaining early permissions for healthcare workers to travel during lockdown periods, which minimized potential disruptions. In parallel, UNHCR relocated some staff to the camps to limit service disruption in the event of a lockdown. Following the nationwide lockdown, small lockdowns were instituted when and where surges of cases appeared. One organization developed rosters of teams that could be mobilized if a localized lockdown inhibited a staff member from traveling to work while permissions were sought. This organization also worked with community-based organizations that could support their patients if a longer lockdown prohibited access and passes could not be granted (this was finally not needed). Some organizations initiated staff transport to facilitate movement in and out of the camps when transport was unavailable during restrictions.

One organization implemented a team structure to maintain staffing in the event of a COVID-19 case among health workers. Healthcare workers were organized into rotating teams of staff that always worked together. When a staff member fell ill, the entire team isolated, and another team was available to fill the gap. One health facility reported hiring three extra general practitioners to staff its clinics 24 hours (a 300% increase); the increased staffing reduced acute referrals to higher-level care facilities and has been maintained since its implementation.

Remote working was introduced according to feasibility. Work-from-home protocols were developed, and new supervisory roles were created to support that. A recurrent enabler to successful working from home was the availability of internet and laptops. In Uganda, one organization supplied internet to its staff to support remote working, which has been continued for some staff. Only limited staff needing to use the office space were permitted to access the premises, and appropriate IPC measures such as distancing, handwashing facilities, and face masks were actioned.

Task shifting was also used to maintain and expand services and reach populations. Most task shifting meant expanding CHW/VHTs’ role to include COVID-19 messaging, surveillance, and vaccination, for example. Where NCD care was moved to the community, CHWs/VHTs assisted with patient follow-up and drug delivery.

Staff with health backgrounds were redistributed to support healthcare delivery, and staff such as drivers who could not work as readily during lockdown periods were redistributed to support administrative tasks.

**Table 3:** Detailed examples of adaptations implemented by organizations across the workforce.

*Workforce adaptations*		
*Increasing staff availability*	Methods to increase the number of staff on shift at any one time	**Jordan** • Increased clinic staff through new hires to maintain services despite staff illness; increased working hours and care for patients who would have typically been referred. • Back up rosters of staff in other locations to fill gaps if staff unable to travel. • Two teams of HCW to allow for easier isolation if positive cases among staff. • Provided staff transport. **Uganda** • Used local surge teams to increase staffing. • Leave policies reduced. • Increase HCW hours. • Deprioritized individual capacity building to free up staff time.
*Task shifting and expansion of community health worker roles*	Expansion of staff roles, particularly CHWs.	**Both countries** • Some of the following tasks were incorporated in existing CHW/VHT roles, with other roles scaled back to accommodate these (such as other ID screening). ◦ Community COVID-19 testing ◦ Support for isolating community members ◦ Community Surveillance expanded to include COVID-19 ◦ ANC, PNC ◦ IYCF screening ◦ Prescription Drug delivery ◦ COVID-19 messaging ◦ Vaccinations (newborn, missing doses, COVID-19) ◦ Nutrition **Jordan** • Repurposed staff with health backgrounds to the facilities. • Drivers and other staff moved to help with administrative tasks and distribution.
*Staff support*	Any adaptions to support staff in their role	**Both countries** • Telephone/internet credit provided for staff. **Uganda** • Support to isolating staff at facilities – e.g., groceries.

*Acronyms used in the table: CHW: community health worker, VHT: Village Health Team, ANC: Antenatal Care, PNC: Postnatal care, IPC: Infection Prevention and Control, PPE: Personal Protective Equipment, HCW: Healthcare worker, IYCF: Infant and Young Child Feeding*

### Medical products, vaccines, technology adaptions

Several informants mentioned initial concerns over delays in procurement and market availability of personal protective equipment (PPE) and medications. In both countries, UNHCR coordinated bulk procurement of PPE for its partners to support the national response. Although some delays were reported in receiving this equipment, this was before any registered cases. Increased costs of some supplies were also a concern, as they led to higher programmatic costs. Some organizations increased local procurement for some supplies. Due to staff movement restrictions in and out of the camps during lockdowns in Jordan, stores were moved inside or very close to the camp boundaries to maintain access to supplies.

Informants frequently discussed adaptations in medication delivery and processes (Table 4). New processes were established to reduce patient volume at health centers and reduce the risk of COVID-19 infection. A typical adaptation was the introduction of multi-month prescriptions for patients with NCDs, HIV, and tuberculosis. The exact implementation varied by organization and location, but it included extending monthly prescriptions to 2–3 months for stable patients alongside new distribution systems. Extended prescriptions and new distribution systems were combined with telephone follow-ups, hotlines for questions, and home visits to maintain patient contact. In some adaptations, CHWs took on additional tasks, including drug delivery and patient follow-up. Outside the camps in Jordan, a delivery company, UPS, was contracted to support home delivery of medication to patients’ homes. Although there were some delays in contracting and data sharing, this delivery mechanism was launched in April 2020. Staff were redistributed from closed clinics to support the packaging of medications at the warehouse, while further permits for staff movement during the lockdowns were acquired. Good electronic record keeping and availability of updated contacts were significant enablers of this.

The limited equipment availability for home-based blood pressure or blood sugar monitoring was a barrier to remote disease management. So was the availability of equipment to maintain the cold chain for insulin Maintaining the 3-month supply was repeatedly raised as a challenge and on one occasion supply and delivery delays led to a brief one time return to one-month prescriptions. Redistribution and pooling of stock between partners took place to minimize over-procurement and limit stockouts.majority reported a return to one-month dispensing or prescriptions in the three years following the start of the COVID-19 pandemic, primarily due to patients’ demand for increased follow-up and to reduce lost medications. A pharmacy in one settlement in Uganda started prepackaging drugs for common conditions, reducing wait and contact time at the dispensing window. This was well received and remains in place.

Organizations also contributed to the national vaccine strategies, ensuring refugee populations were included and supporting vaccine delivery using CHWs to deliver door-to-door vaccines, engage and sensitize communities, and reduce vaccine hesitancy. In Jordan, several organizations provided cold chain assistance to the MoH vaccination campaign using their existing measles cold chain and facilitated registration and transport to vaccination points. One organization set up mobile vaccination clinics to provide vaccinations to refugees living in camps struggling to access them. The mobile clinic would distribute one COVID-19 vaccination type daily to avoid confusion. MoH vaccinators and their messaging platform would inform individuals about vaccine distribution schedules.

Informants mentioned difficulties brought about by the long waits to obtain PCR results early in the pandemic, as tests needed to be processed centrally or regionally. The early availability of RDTs in late 2020 and early 2021, coupled with clear testing protocols, was described as a significant step in better identifying COVID-19 cases. UNHCR HQ-level negotiations with testing companies facilitated rapid delivery of RDTs shortly after their approval. One organization reported having access to RDTs even before MoH guidance on their use was available in Uganda. Such early access caused some uncertainty while waiting for government guidance on testing and isolation to be updated.

**Table 4:** Detailed examples of adaptations implemented by organizations across medical products vaccines and technologies.

Medical products, vaccines, technologies adaptions		
*Procurement*	Novel procurement mechanisms	**Both countries** • Bulk procurement of PPE by UNHCR for partners. **Jordan** • Procurement of ventilators to support national response. • Pooling and redistribution of stock • Early stock checks to identify potential gaps. **Uganda** • Increased local procurement of PPE.
*Medicine management*	Adaptions in medicine management from warehouse to delivery to reduce face to face contact with patients and hence transmission	**Both countries** • Multi-month prescribing and dispensing. **Jordan** • Contracted UPS and private pharmacies for drug delivery to stable patients with chronic illnesses. • CHW drug delivery. • Warehouses moved into camp bounds to ensure access. • Redistributed staff to support prescription preparation for delivery. **Uganda** • Prepacked common drug regimes. • Advocating for longer prescriptions and generics to reduce financial burden. • Staff dropping off prescriptions to patients.
*Supporting national COVID-19 vaccination strategies*	Adaptations to support the national vaccination strategies	**Both countries** • Community engagement to understand barriers to COVID-19 vaccination **Jordan** • Supported MoH vaccination cold chain. • COVID-19 vaccination mobile clinics.
*Building testing capacity*	Adaptations to build testing capacity and build on local testing capacity	**Jordan** • Specified testing and treatment areas. • Early move to RDTs **Uganda** • Early agreement at HQ level for RDTs negotiated and quickly disseminated.

*Acronyms used in the table: CHW: community health worker, RDTs: Rapid Diagnostic Tests, MoH: Ministry of Health;*

### Health information system adaptations

The addition of COVID-19 indicators to health information systems represented the main adaptation in this domain (Table 5). Sound pre-existing health information systems were instrumental for this and other adaptations. Where existing systems were not up-to-date, additional efforts were needed to ensure patients were not lost to follow-up, including using social media to encourage patients to contact clinics for follow-up visits and manually searching paper records to find contact information. Increased awareness about the importance of patient record completeness led to the introduction of checks to ensure records were up-to-date during subsequent interactions. In one instance, an electronic clinical management system was due to be introduced in 2022, and its arrival facilitated improved remote patient management.

### Funding/Financing adaptions

Informants did not report funding challenges specific to the COVID-19 pandemic. Limited funding for programs was an ongoing challenge; however, existing preparedness plans following prior epidemics, increased funding availability from donors for the response, and careful costing were reported as enablers to access required funds and allow rapid procurement of supplies, in particular, PPE, as well as providing the latitude to develop new adaptations (Table 5).

### Leadership and governance adaptations

Early planning, internal continuation policies, prior preparedness plans, and clear messaging to staff were instrumental in maintaining services (Table 5). Strong leadership and governance were essential enablers of timely adaptation and coordination throughout the COVID-19 pandemic. Strong support from the HQ level (clear guidelines, webinars, and mentorship through online platforms) enabled programmatic adaptation and increased confidence among staff. One organization supported attendance by supplying field staff with internet facilities.

Regarding coordination and continuation of activities, coordination meetings were moved online. In Jordan, the UNHCR camp focal point moved to the camp to lead the response (anticipating possible movement restrictions). Rapid response teams were also activated early on, chaired by MoH and UNHCR, and their partners were invited to participate. They had two main roles: setting up service continuity plans and ensuring adequate supplies. Technical working groups were established to provide COVID-19 guidance.

In Uganda, a task force was created at the national level to provide guidance, communicate activities, and support the division of tasks across sectors. Further task forces were set up at various levels (for example, at the district level) to coordinate and implement adaptations. District task forces were primarily responsible for the implementation and training of IPC measures, case management, and surveillance. Collaboration across stakeholders at district and community levels improved thanks to the task forces. Village health task forces ensured community involvement and reduced the top-heaviness of the response. Their role looked different across communities and over time, but it included surveillance, risk communication, and the identification of new arrivals to support quarantining and testing.

The role of leadership in advocating for the populations served was also emphasized. Organizations worked with the MoH and WHO to ensure the integration of refugees into national responses, for example, within the national vaccine plan in Jordan. Including UNHCR in the government response in Jordan ensured MoH COVID-19 messaging reached refugee populations. It also facilitated the coordination with government health facilities for testing, referral, and vaccination. Pre-existing established relationships between the MoH, UNHCR, and their partner organizations were vital for an effective and efficient response.

**Table 5:** Detailed examples of adaptations implemented by organizations across health information systems, funding and financing and;leadership and governance.

**Health information system adaptations**	Actions to adapt or improve health information systems	**Jordan** • Used social media to reach patients whose details were not available in existing medical records to be able to provide telephone follow-up and drug delivery. • Accelerated electronic medical record implementation to allow better remote working and information sharing. **Uganda** • Used existing HIS service to identify drops in service usage and support access in those areas
**Funding/ Financing adaptions**	Actions to ensure sufficient funding for the response	**Both countries** • Early requests for increased funds. **Uganda** • Early costing. • Existing epidemic contingency plans.
**Leadership and Governance adaptations**	Any change in how leadership acted and in governance including national policy changes	**Jordan** • Camp focal point moved to camp. • Clear messaging and preparedness measures introduced for anticipated lockdowns. • Advocacy for inclusion of refugees in national guidance. • Business continuity plans updated. • Rapid response team enabled. • Technical working group developed to support response. • PH focal points granted permission to stay in the camps. **Uganda** • Modification of National COVID-19 guidance to meet needs of refugees in settlements. • Adaption of existing continuity plans to COVID-19 context. • HQ developed support for education and adaptations. • Development of new guidelines. • Development of National, District, and Village health taskforces to better integrate, communicate, and coordinate the response.

*Acronyms used in the table: HIS: Health Information System, PH : Public Health.*

## Discussion

This paper demonstrates the numerous and comprehensive innovative adaptations implemented across the entire health system to maintain health services for refugees in Uganda and Jordan during the COVID-19 pandemic. Throughout all the interviews, the creativity and drive by staff to support continued services with minimal disruptions while keeping patients and staff safe was consistent and tremendous. Adaptability is an important tenant of a resilient health system [31–34]. Through taking a health system lens, we highlight the breadth of adaptation that was needed across services and the resilience of healthcare staff, leadership, and the system to dynamically adapt to various challenges caused by the COVID-19 pandemic to ensure continued access to care for refugees in different contexts. The COVID-19 pandemic created an environment where innovation and adaptation were needed to address new and changing health needs within existing resource constraints [35]. These adaptations extended beyond simple IPC measures to reduce immediate infection spread. They included ways to keep the public informed, facilitate access to care during lockdowns and curfews, and change health service delivery modalities to sustain utilization. Strategies often focused on immediate problem-solving; however, some, such as the new appointment systems or prepackaging of medication for routine conditions, were reported to improve functioning and remained. Understanding the adaptations that were successful and what facilitated their success is important for improving healthcare delivery and preparedness for future pandemics.

Identifying and fostering factors that enable adaptations is key for future responses. In our study, key facilitators in supporting nimble and responsive adaptations were evident throughout the analysis. These included 1) integration of refugees in National Health systems, 2) strong relationships and a supportive environment, 3) existing preparedness plans and 4) financing.

Uganda and Jordan provide a specific context where refugees are integrated into existing host countries’ health plans and services [36,37]. In Jordan, refugees were included in the COVID-19 National Preparedness and Response Plan, which defined access to testing, vaccinations, and treatment for the entire society (in and out of camp) [38]. Syrian refugees have been integrated into national health services at non-insured Jordanian rates since 2019; non-Syrian refugees were included in 2020 [36]. Uganda’s Health Sector Integrated Refugee Response Plan incorporates refugees in settlements into the MoH’s national services which encompass routine, emergency services as well as outbreak preparedness and response [37]. Vaccination and testing adaptations were mentioned less than other adaptations. This is likely due to the inclusion of refugees within national response plans and health services in each country and the enabling environment, which allowed organizations to leverage national capacity and supplies instead of requiring a parallel system for refugees. The integration of the refugees into the national systems led to a more efficient response that avoided duplication of systems and could benefit from the existing network of health facilities in the two countries. Such a parallel system would have likely delayed and reduced consistent access where global resources were limited.

UNHCR and its partners, thanks to their existing relationships and longstanding presence, were able to advocate for refugee inclusion in national plans and work within these response structures, supporting the government’s response and adapting guidance to meet the needs of the entire population. This strong governance was also evidenced through early bulk PPE purchasing for partners, proactive early contracts for RDTs, and pooling of drugs at risk of stock-out, which limited disruptions to care. Good governance is an essential cross-cutting component of a resilient health system that can absorb, adapt, and transform in response to shocks [39,40]. All interviews discussed the importance of guidance from MoH, UNHCR, or their own HQ to support response adaptations. These supportive environments were essential in facilitating and enhancing innovation.UNHCR’s leadership and coordination were repeatedly described as enablers of adaptations across the health care system. The strong relationships between partners were further evidenced during the vaccine roll-out. A willingness of all partners to work together was evident and reduced delays. Differences in the adaptation strategies described by NGO and UN partners were not eveident through the interviews, rather the relationships described appeared to support an alignment of action that assisted the implementation of adaptations. UNHCR and partners reciprocally helped each other and the response, stepping in when resources and capacity were available. For example, organizations provided equipment and staffing to support the national vaccination rollout in both countries.

In this context, financing was not as limiting as it often is. The rapid availability of funds from donors, early costing and existing contingency and preparedness plans allowed rapid distribution of funds to support the development of adaptions and allowed for rapid and bulk procurement, workforce bolstering and infrastructure, which made many of the adaptations viable and successful. Learning from previous epidemics was apparent. Such learning should continue to be documented and integrated into preparedness structures. In Uganda, the Village Health taskforces connected communities to health services and maintained government guidance and regulations, adapting as needs changed and cases grew. Their role expanded since their use during previous Ebola outbreaks, enabling greater community integration and addressing some top-heaviness in the response.

Modifications in care delivery were a common adaptation, and while care delivery for COVID-19 patients was discussed, the majority were to maintain access to care for all patients and attempt to limit the disruption caused by potentially increasing volumes of COVID-19 patients and restrictions as part of the in-country public health response. Many of the innovations described aligned and expanded on those previously mentioned in the literature [14–16]. Adaptations to NCD care were a frequently mentioned area of rapid adaptation. Packages of interventions were designed to move care to the community and limit the need and frequency of clinic attendance for stable patients by extending prescription length, facilitating drug delivery, and introducing telephone follow-up. Similar COVID-19 adaptations for NCD patients have been documented across IRC programs in Kenya, Thailand, Somalia, Uganda, and Jordan [16]. These changes were described as well-received by service users in our interviews. However, issues with supply and medication loss were reported, and services had returned to previous prescription lengths and delivery systems for NCDs. Nonetheless, such a package of interventions to maintain access to care for patients has potential utility in future pandemics. Further research is needed on the impact of such interventions in these settings on adherence, engagement and outcomes, which may offset concerns about medication loss [41]. Other particularly innovative adaptations in care delivery included the prepackaging of common medication regimes, which has been continued and improved efficiency, the use of reverse referrals to support access to specialist care in lower-level treatment centres and the integration of malaria RDTs within triage pathways to help support the differentiation of causes of fever on initial assessment.

CHW’s expanding and varied roles were evident in both contexts and supported a multitude of adaptations in care delivery. CHW/VHTs were well positioned to have a flexible scope and be quickly trained to respond to changing needs, playing an integral role in bridging the gap between the community and the health facilities. This vital role was also identified by Ratnayake et al. in their paper, where CHWs’ role expanded to support Syrian refugee NCD patients with medication, self-management, and psychosocial support. Enrolled patients did not experience worsening health status, and CHW helped identify COVID-19 cases [15]. CHWs are an important asset in many countries, and their roles are adapted to provide critical support to COVID-19 responses in multiple contexts [42–45].

Technology played a larger role in innovations in Jordan where existing capacity supported a smoother transition to remote working and coordination. Technology was incorporated in packages of intervention and included the use of mobile apps, telephone services and social media to support care delivery, follow-up, referrals, psychosocial support, community engagement and risk communications. HIS were an important enabler in monitoring and evaluation. In LMIC contexts, more generally, telemedicine was a frequent focus of many health system adaptations during COVID-19 [46,47]. Similarly, many adaptations described here used telephone services such as hotlines for health workers and patients and telephone check-ups (to confirm medication receipt and educate on disease management or COVID-19). However, less frequently did this encompass full patient consultations as often described, particularly in Uganda. Improved infrastructure and user access are needed to support technology in playing a greater role in adaptations in the future.

Existing work has demonstrated the likely positive impacts of the adaptations described to limit the impact of COVID-19 and maintain healthcare access early in the pandemic. Previous research in Jordan showed reduced COVID-19 transmission in camps compared to out-of-camp during the first year of the pandemic and some immediate declines in health service usage in camps differing across services, which normalized over time [8]. Similarly, in Uganda, a slight drop in acute health care utilization was identified in settlements, but preventative care usage remained consistent [7]. In both cases, infection rates and mortality secondary to COVID-19 were less than anticipated as were interruptions to existing services [7,8]. Appreciating and understanding the adaptations made in both contexts where the early impact was less than expected is important for learning, health system strengthening, and planning for future pandemics. Understanding adaptations in camp/ settlement scenarios is vital, as while implementation of regulations may appear more manageable, creativity in adaptation was even more critical in such controlled environments.

### Limitations

This study has limitations. First, we were not able to interview members of the Ministry of Health in Uganda or Jordan despite multiple attempts to make contact via UNHCR. While we spoke to a wide array of individuals across organizations and levels, we might have missed some adaptations or perspectives. Yet, as the refugee response is integrated in the national systems, partner institutions describing their adaptations indirectly described the national response. Secondly, reporting the exact number of adaptations described by participants was challenging due to the variation in how these were defined, from individual actions to packages of interventions or adaptations and therefore not included. Thirdly, the performance of individual adaptations in reducing COVID-19 infection or maintaining access to services was not evaluated as this was outside the study’s scope. While the efficacy of many interventions to reduce COVID-19 spread can be assumed from our understanding of COVID-19 infection dynamics, it is not possible to assess the individual impact of these interventions. Lastly, we did not investigate how the changes impacted service use and quality of care. We are currently conducting work that includes an assessment of perceptions of changes made to health care over this time in refugee settings in both Uganda and Jordan.

## Conclusion

This study highlights the breadth and scope of innovative adaptations made to maintain health services for refugees in Jordan and Uganda. It demonstrates the resilience and creativity of staff working in contexts where refugees are integrated into national response plans and health care delivery. Learning from these adaptations, their enablers, and barriers can inform preparedness and response strategies to future outbreaks in forced displacement settings. Furthermore, many of these adaptations continue to be used beyond the pandemic as they improve the overall health system. These adaptations should be further explored to understand how they could contribute to continued health service delivery in other complex scenarios. Evaluating these adaptations in future responses will help estimate their effectiveness and improve their implementation.

## Supporting information

S1 Table Table summary of the adaptations disussed. (DOCX)

S1 Text In-depth interview guide. (DOCX)

S2 Text Simplified interview guide. (DOCX)
