# Supplementary material for: Strategies to maintain health service provision during the COVID-19 pandemic in refugee settings in Jordan and Uganda
Source: PLOS Glob Public Health. 2025 May 8;5(5):e0004484. doi: 10.1371/journal.pgph.0004484 (PMC12061133; doi:10.1371/journal.pgph.0004484)
Supplement: S1 Table — (DOCX) [file pgph.0004484.s001.docx]

**Annex 1:**

A complete summary of discrete adaptations mentioned by respondents by health building block

| Adaptation theme | Explanation | Country examples |
| --- | --- | --- |
| **Health service delivery adaptations** | | |
| *Procedures for screening and isolation* | Facility- and camp-level processes to screen and isolate potential cases. | **Jordan**   - Defined areas in camps for isolation and quarantining, with separate services including food, sanitation - Quarantine area near health facility with monitoring from the health facility for more severe cases. - Plot-based isolation for community cases in camps. - Introduced individual kitchens and latrines for households. - Hotline for isolating individuals needing support. - House-to-house surveillance.   **Uganda**   - Zoning: cohorting new arrivals in smaller transit centers for joint quarantine. - Community identification and screening of new arrivals. - Isolation units built for suspected cases. - Toll-free line for VHTs and community leaders to support surveillance communication. |
| *Adapted triage* | Adaptations to triage processes to identify potential COVID-19 cases and those needing immediate treatment. | **Both countries**   - Temperature screening and case definition screening on arrival to health facilities.   **Jordan**   - Separate secondary triage for suspected cases.   **Uganda**   - Malaria RDTs moved to consultation rooms/ isolation areas to identify other causes of fever and limit patient movement to the laboratory. - Identified isolated beds ready to care for critically unwell COVID-19 patients on arrival. |
| *Case management and Referral pathways* | Referral mechanisms to provide care for COVID-19 patients, reduce referral burden on secondary and tertiary care level facilities, as well as maintain care for patients that would have ordinarily been referred | **Jordan**   - Designated MoH facility for urgent non-covid referrals, new referral mechanisms into MoH facilities for non-COVID-19 cases. - WhatsApp service to upload requests for and receive reimbursements for MoH facility care. - Newly built specific COVID-19 treatment centers to manage cases.   **Uganda**   - Toll-free number for COVID-19 treatment advice and referral from local facilities to designated COVID-19 secondary care facilities. - Delayed cold-case referrals to tertiary centers. - Reverse referrals, specialists visited the lower-level health centers. - All COVID-19 patients initially referred to the capital, subsequently to a subregional center. - Redistributed patients regionally to manage Oxygen availability. - COVID-19-specific ambulances: to pick up patients in the community and transfer them to treatment centers. |
| *Reducing face-to-face contact* | Reduction of face-to-face appointments to reduce the risk of contact transmission and free up health care workers to manage COVID-19 cases | **Both countries**   - Multi-month prescribing (NCD, TB, HIV, Nutrition, FP). - Health care providers conducting telephone consultations for stable patients with chronic illnesses (NCD and some gynecological care). - Outreach mobile care for ANC and PNC. - Home visits for high-risk, unstable, or newly diagnosed patients with chronic illnesses.   **Jordan**   - Mobile teams for acute cases, cases flagged by CHWs. - Positive cases telephone follow-up. - Hotlines available for NCD patients to receive advice. - Appointment system introduced. - Patient transportation system to facilitate movement to the clinic. - Limited certain services, including dental care, newborn screening, growth monitoring. - Reduction of patients seen in the clinic per day. - Community outreach by CHWs to NCD patients. - Telephonic psychological support services.   **Uganda**   - Earlier clinic opening, to reduce morning crowds. - Limited clinic time to half a day. - Reduce face-to-face appointment frequency for clinically stable patients with chronic illnesses. - Reduced frequency and contact time of house-to-house VHT visits. |
| *IPC measures* | Simple processes introduced to reduce infection transmission | **Both countries**   - Provided masks to patients and visitors. - Limited visitors to health facilities. - Reorganized waiting areas, new seating for physical distancing, separated waiting areas for suspected cases. - Earlier opening, later closing and increased time between appointments for cleaning. - Dedicated clinic spaces for patients meeting the case definition. - Increase handwashing facilities. - Increased spacing between beds.   **Jordan**   - Dedicated facility pharmacies for suspected cases. - Staff to manage crowds at the health facilities. - Different entry and exit points in the facility for suspected cases. - Random staff testing.   **Uganda**   - IPC volunteers recruited and trained to institute and maintain IPC measures at facilities. |
| *COVID-19 community support* | Methods to inform the public about COVID-19 and to support health care seeking in the community | **Both countries**   - COVID-19 advice hotlines.   **Jordan**   - Social media messaging on COVID-19 and health facility changes. - Target CHW visits to community members vulnerable to COVID-19. - Posters and flyers. - Change in cash for health services with greater routes for accessing funds including the introduction of e-wallets, expansions of Iris scanning schemes and home delivery . - CHW delivery of supplies such as masks along with messaging.   **Uganda**   - Repurposing other vehicles for health to collect and bring community members to the health facility. - VHTs met the public in small groups to explain HS changes, IPC, and COVID-19. - Recorded health messages disseminated through speakers on motorbikes. - VHTs with megaphones to disseminate information. |
| ***Workforce adaptations*** | | |
| *Increasing staff availability* | Methods to increase the number of staff on shift at any one time | **Jordan**   - Increased clinic staff through new hires to maintain services despite staff illness; increased working hours and care for patients who would have typically been referred. - Back up rosters of staff in other locations to fill gaps if staff unable to travel. - Two teams of HCW to allow for easier isolation if positive cases among staff. - Provided staff transport.   **Uganda**   - Used local surge teams to increase staffing. - Leave policies reduced. - Increase HCW hours. - Deprioritized individual capacity building to free up staff time. |
| *Task shifting and expansion of community health worker roles* | Expansion of staff roles, particularly CHWs. | **Both countries**   - Some of the following tasks were incorporated in existing CHW/VHT roles, with other roles scaled back to accommodate these (such as other ID screening).   - Community COVID-19 testing   - Support for isolating community members   - Community Surveillance expanded to include COVID-19   - ANC, PNC   - IYCF screening   - Prescription Drug delivery   - COVID-19 messaging   - Vaccinations (newborn, missing doses, COVID-19)   - Nutrition   **Jordan**   - Repurposed staff with health backgrounds to the facilities. - Drivers and other staff moved to help with administrative tasks and distribution. |
| *Staff support* | Any adaptions to support staff in their role | **Both countries**   - Telephone/internet credit provided for staff.   **Uganda**   - Support to isolating staff at facilities – e.g. groceries. |
| **Medical products, vaccines, technologies adaptions** | | |
| *Procurement* | Novel procurement mechanisms | **Both countries**   - Bulk procurement of PPE by UNHCR for partners.   **Jordan**   - Procurement of ventilators to support national response. - Pooling and redistribution of stock - Early stock checks to identify potential gaps.   **Uganda**   - Increased local procurement of PPE. |
| *Medicine management* | Adaptions in medicine management from warehouse to delivery to reduce face to face contact with patients and hence transmission | **Both countries**   - Multi-month prescribing and dispensing.   **Jordan**   - Contracted UPS and private pharmacies for drug delivery to stable patients with chronic illnesses. - CHW drug delivery. - Warehouses moved into camp bounds to ensure access. - Redistributed staff to support prescription preparation for delivery.   **Uganda**   - Prepacked common drug regimes. - Advocating for longer prescriptions and generics to reduce financial burden. - Staff dropping off prescriptions to patients. |
| *Supporting national COVID-19 vaccination strategies* | Adaptations to support the national vaccination strategies | **Both countries**   - Community engagement to understand barriers to COVID-19 vaccination   **Jordan**   - Supported MoH vaccination cold chain. - COVID-19 vaccination mobile clinics. |
| *Building testing capacity* | Adaptations to build testing capacity and build on local testing capacity | **Jordan**   - Specified testing and treatment areas. - Early move to RDTs   **Uganda**   - Early agreement at HQ level for RDTs negotiated and quickly disseminated. |
| **Health information system adaptations** | Actions to adapt or improve health information systems | **Jordan**   - Used social media to reach patients whose details were not available in existing medical records to be able to provide telephone follow-up and drug delivery. - Accelerated electronic medical record implementation to allow better remote working and information sharing.   **Uganda**   - Used existing HIS service to identify drops in service usage and support access in those areas |
| **Funding/ Financing adaptions** | Actions to ensure sufficient funding for the response | **Both countries**   - Early requests for increased funds.   **Uganda**   - Early costing. - Existing epidemic contingency plans. |
| **Leadership and Governance adaptations** | Any change in how leadership acted and in governance including national policy changes | **Jordan**   - Camp focal point moved to camp. - Clear messaging and preparedness measures introduced for anticipated lockdowns. - Advocacy for inclusion of refugees in national guidance. - Business continuity plans updated. - Rapid response team enabled. - Technical working group developed to support response. - PH focal points granted permission to stay in the camps.   **Uganda**   - Modification of National COVID-19 guidance to meet needs of refugees in settlements. - Adaption of existing continuity plans to COVID-19 context. - HQ developed support for education and adaptations. - Development of new guidelines. - Development of National, District, and Village health taskforces to better integrate, communicate, and coordinate the response. |

*Acronyms used in the table: CHW: community health worker; VHT: Village Health Team; RDTs: Rapid Diagnostic Tests; MoH: Ministry of Health; NCD: Non-communicable diseases; TB: Tuberculosis; HIV: Human Immunodeficiency Virus; FP: Family Planning; ANC: Antenatal Care; PNC: Postnatal care; IPC: Infection Prevention and Control. PPE: Personal Protective Equipment, HCW: Healthcare worker, RDTs: Rapid Diagnostic Tests, MoH: Ministry of Health, IYCF: Infant and Young Child Feeding, HIS: Health Information System*
