# Supplementary material for: Strategies to maintain health service provision during the COVID-19 pandemic in refugee settings in Jordan and Uganda
Source: PLOS Glob Public Health. 2025 May 8;5(5):e0004484. doi: 10.1371/journal.pgph.0004484 (PMC12061133; doi:10.1371/journal.pgph.0004484)
Supplement: S1 Text — (DOCX) [file pgph.0004484.s002.docx]

**Health service delivery and program adaptations in refugee settings during the COVID-19 pandemic**

**Key Informant Interview guide – IRB 00023096**

| **BACKGROUND INFORMATION** |  |
| --- | --- |
| KII date |  |
| Interviewer |  |
| KII number |  |
| **CONSENT** | |
| Good morning/afternoon/evening. I'm .......... from Johns Hopkins University. We are conducting a study in collaboration with the United Nations High Commissioner for Refugees. The purpose is to learn more about the adaptations made to healthcare services in Jordan/Uganda, throughout the COVID-19 pandemic, including during lockdowns and other issues that arose throughout the pandemic. If you agree to participate, we would like to talk to you in your professional capacity as _[role/position]_______. We will ask you questions about the healthcare service adaptations your organization and others made during the COVID-19 pandemic and the processes involved in doing them. We are interested to learn about which changes have been made, which have been successful, which were less so, and why. We would also like to discuss which changes are still in place and which could be useful for future outbreak responses. We hope the information gained will help to better understand strategic and operational adaptations to health service delivery that aimed to maintain essential health services for refugees inside and outside of the camps.  Participation in this interview is voluntary and will take about 60 minutes. If at any point there are any questions you do not feel comfortable answering, you can choose not to answer them. You can also choose to stop the interview at any point. Your answers will be kept confidential and no reference will be made to any individual respondent. Your answers will be accessible only to the research team.  Do you need any further information or do you have any questions?  If you have questions or if you feel you have not been treated fairly, please feel free to contact the study coordinator (Name) at this number (XXX).  Do you agree to participate in this interview? Yes / No (END INTERVIEW)  Do you agree that we record this interview (to facilitate taking notes)? Yes / No (DO NOT RECORD) | |

Before starting:

Can you confirm your position in the organization ______________________

How long have you been working here for your organization (in months/ years) [*circle the unit the applies and note the number*]? ________________

**To start, we would like to clarify the objectives and the scope of the interview**.

We would like to **know more about how COVID-19 has changed your organization's health programming** from the beginning of the pandemic and how things have evolved over time.

We are interested in better understanding how you addressed challenges brought about by the COVID-19 pandemic **by changing or adapting the way you operated** in refugee settings. For changes or adaptations, we mean for example

- the introduction of telemedicine (where previously not used), or the expansion to other services
- how triage was adapted to include respiratory infections symptoms
- the introduction of infection prevention and control measures in health facilities and how patient flow, or facility organization changed
- the expansion of existing or the creation of new wards (for example to treat covid patients)
- the change of hours/ duration or frequency of services
- whether human resources were repurposed and how; task shifting
- changes in drug prescription / delivery
- changes in where the service was delivered.

We are interested in the **broad range of services** your organization offered, from preventive to curative (both COVID and other conditions), for children or adults, delivered in health facilities, mobile clinics, in the community, or remotely. Depending on the number of changes/adaptations that were introduced, we will select the ones to focus on.

We would like to **retrace with you the various phases of the pandemic** and document how challenges, solutions, constraints and enabling factors evolved.

We hope to hear from you what was **unique to COVID that you think should continue or could be useful for other outbreak responses**.

1. First, we would like to define the periods that characterized the pandemic in your context.
   1. Could you recall different phases? For example, were there phases that you could define as “Intense”, “Less intense”, and “’post’ COVID”
   2. Let’s try establishing when they started and ended, and by what they were characterized

|  | Start (date) | End (date) | Key features |
| --- | --- | --- | --- |
| Period 1 |  |  |  |
| Period 2 |  |  |  |
| Period 3 |  |  |  |
| XXX |  |  |  |

Using the periods identified above, we would like to better understand how the pandemic influenced your operations.

|  | Period 1 [defined as above] | Period 2 [defined as above] | Period 3 [defined as above] |
| --- | --- | --- | --- |
| 1. Which challenges did you face? (HR, funding, access to population, procurement, movements, others?) |  |  |  |
| 1. Did you introduce changes to maintain health service provision?   (it could be both in response to certain challenges, or to anticipate possible problems)  *If yes, go to 4*  *If no, go to 16* |  |  |  |
| We would like more details about X of the X changes you mentioned. Let’s start with #1 |  |  |  |
| 1. Can you describe the change/ adaptation (content) |  |  |  |
| 1. Which health service were affected with this change? |  |  |  |
| 1. Did the change relate to the format /delivery modality? |  |  |  |
| 1. Did the change relate to hours of operation of the facility, or frequency / duration a service was provided? |  |  |  |
| 1. Did the change relate to the setting / location where the service was provided?   (home/ facility/ mobile) |  |  |  |
| 1. Any change related to the personnel who delivered the service? |  |  |  |
| 1. What about the target population? |  |  |  |
| 1. Who decided on /defined the change/adaptation? |  |  |  |
| 1. Can you specify when this change was introduced? (Starting date) |  |  |  |
| 1. Can you specify when this change was stopped? (Ending date) |  |  |  |
| 1. Could you tell us why it was stopped? Or why it hasn’t been stopped? |  |  |  |
| 1. Any other notes: |  |  |  |
|  |  |  |  |

*[Repeat questions 4 to 15 for the other changes/ adaptations; add rows to the table]*

|  | Period 1 [defined as above] | Period 2 [defined as above] | Period 3 [defined as above] |
| --- | --- | --- | --- |
| 1. Can you tell us more about **human resources**?    1. Did you have all human resources you needed?       1. Did you experience shortages of HR? (ex bc of illness or fear of COVID)       2. How did this affect the changes/adaptations implemented?    2. How did you adjust if you had fewer or more people than needed?       1. Were people repurposed?       2. Did you have to modify their job description (task shifting)?    3. Did anything change in the profile(s) of the workforce? |  |  |  |
| 1. Could you procure sufficient **material and equipment** for what was needed?    1. How did this affect the implementation of the changes/adaptations that you discussed above?    2. How did you adjust if you could not procure all needed equipment?   *If yes (i.e. no issue with procurement), go to 18* |  |  |  |
| 1. Did you have sufficient **funding** to implement these adaptations?    1. How did this affect the implementation of the changes/adaptations that you discussed above?   [*If funding depended on the adaptation, ask for each change/adaptation discussed above*] |  |  |  |
| 1. Which **challenges** did you face in implementing the program changes/adaptations?   [*If challenges depended on the adaptation, ask for each adaptation discussed above*] |  |  |  |
| 1. Were there any factors that **facilitated** the implementation of the program changes/ adaptations?   *Prompt: factors may be contextual; at the organization level; related to leadership / personality; characteristics of the provider; characteristics of the adaptation.*  [*If enabling factors depended on the change/adaptation, ask for each adaptation discussed above*] |  |  |  |
|  |  |  |  |

We would like now to discuss if the changes/adaptations **worked as planned**, meaning were they **effective** and produced the **expected outcomes**.

1. Were some changes/adaptations **more effective** than others and why?
2. Were there any changes/adaptations that were attempted at the start of the COVID-19 pandemic that **didn’t come to fruition or were stopped early**, and if yes, why?
3. Have these changes/adaptations been **monitored/ evaluated**? Y/N

If yes:

- 1. How?

*Prompt: qualitative vs quantitative; process, outcome or impact indicators.*

- 1. Were they any lessons learned?
  2. Could you share with us evaluation or monitoring reports?

If no:

- 1. Why?

1. Are any of the changes/adaptations going to be or should be **maintained** beyond COVID-19?
   1. Why?
   2. Are any of the changes/adaptations made that are still in operation?
   3. Are there any that you think should be maintained beyond the COVID-19 context as it could be helpful for other outbreak response or emergency settings?
2. Looking back if you were faced with the COVID-19 pandemic again, what would you **do differently?**

**We would like to know more about how you introduced the changes and adaptations to the communities where you work and how they were received.**

1. How were the changes/adaptations accepted by the **communities**?
   1. Were any changes/adaptations more accepted than others, and if yes, why?
2. Was there a consultation process when changes/adaptations were introduced? (for example, with health staff, with community members, community leaders, or groups of patients?
3. How were the changes communicated?

**We have reached the end of the interview**

1. Is there anything else you would like to share with us ?

Thank you for your time.

Can we get back in touch with you if we need to confirm some details?
