# Supplementary material for: Strategies to maintain health service provision during the COVID-19 pandemic in refugee settings in Jordan and Uganda
Source: PLOS Glob Public Health. 2025 May 8;5(5):e0004484. doi: 10.1371/journal.pgph.0004484 (PMC12061133; doi:10.1371/journal.pgph.0004484)
Supplement: S2 Text — (DOCX) [file pgph.0004484.s003.docx]

**Health service delivery and program adaptations in refugee settings during the COVID-19 pandemic**

*Key Informant Interview guide*

| **KEY INFORMATION** |  |
| --- | --- |
| Date |  |
| Organization |  |
| Position in the organization |  |
| Since when /how long |  |

**Objectives and scope of the form & interview**

We would like to **know more about how COVID-19 has changed your organization's health programming** from the beginning of the pandemic and how things have evolved over time.

We are interested in better understanding how you addressed challenges brought about by the COVID-19 pandemic **by changing or adapting the way you operated** in refugee settings. For changes or adaptations, we mean for example

- the introduction of telemedicine (where previously not used), or the expansion to other services
- how triage was adapted to include respiratory infections symptoms
- the introduction of infection prevention and control measures in health facilities and how patient flow, or facility organization changed
- the expansion of existing or the creation of new wards (for example to treat covid patients)
- the change of hours/ duration or frequency of services
- changes in community based activities
- whether human resources were repurposed and how; task shifting
- changes in drug prescription / delivery
- changes in where the service was delivered.

We are interested in the **broad range of services** your organization offered, from preventive to curative (both COVID and other conditions), for children or adults, delivered in health facilities, mobile clinics, in the community, or remotely.

We would like to **retrace with you the various phases of the pandemic** and document how challenges, solutions, constraints and enabling factors evolved over time.

We hope to hear from you what was **unique to COVID that you think should continue or could be useful for other outbreak responses**.

Thanks for answering the questions below. We look forward to discussing with you your experience in the interview we just scheduled. Feel free to get in touch with XX or XX if you have any questions or concern.

1. Can you describe the situation at the beginning of the COVID-19 pandemic in terms of new conditions, challenges such as movement restrictions, access to population, human resources, etc?
2. How were your activities impacted by COVID-19 and by the introduced restrictions?
   1. Did you have to change the way you were working?
   2. Can you describe the modifications that your organization introduced?

*Thanks for describing how the modification worked, where it was implemented, whether it applied to several health services, whether it was about how the service was provided, where or by whom?*

- 1. When were these changes introduced and for how long were they maintained?
  2. Did things change over time, according to different phases of the pandemic?
  3. Were the modifications effective?
  4. Are some of these modifications still in place? if not, would you wish they were?
  5. Were the same modifications used in other events (for example during Ebola)? Could they be used in future outbreak responses?
  6. Were any of these modifications monitored or evaluated?

1. Did you have any challenges related to:
   1. Human resources?
      1. For example, did you experience shortages? Did you need surge teams? If yes, why?
   2. Procurement of materials and equipment (PPE, oxygen, other medical material, plastic sheets, etc)
   3. Funding
2. How did these challenges affect your operations? How did you adjust?
3. Were there different phases during the pandemic? Were your operations affected differently at different times?
4. Were there factors that facilitate the implementation of the response?

*These could be both within your team, organization, country level, between organizations, etc*

1. Looking back, would you do anything different?
2. How did you interact with the communities about the adaptations? How did you communicate the changes to them? How did they react? Were they somehow involved in the design or implementation of the changes?
3. Is there anything else you would like to share with us ?

Thank you for taking the time to respond to these questions.

We look forward to discussing with you in the interview we scheduled.
